# *α*_1A_-Adrenergic receptor (*ADRA1A*) signaling signatures as prognostic biomarkers in liver hepatocellular carcinoma: a bioinformatic analysis

**DOI:** 10.1007/s40203-026-00706-4

**Published:** 2026-07-31

**Authors:** Yarely Mabell Beltrán-Navarro, José Vázquez-Prado, Guadalupe Reyes-Cruz, Jesús Adolfo García-Sáinz

**Affiliations:** 1 Department of Cell Biology and Development, Instituto de Fisiología Celular, Universidad Nacional Autónoma de México, Ciudad Universitaria, Mexico City, 04510 Mexico; 2 Department of Pharmacology, Av. Instituto Politécnico Nacional 2508, Col. San Pedro Zacatenco, Mexico City, 07360 Mexico; 3 Department of Cell Biology. Cinvestav-IPN, Av. Instituto Politécnico Nacional 2508, Col. San Pedro Zacatenco, Mexico City, 07360 Mexico

**Keywords:** *ADRA1A*, *ADRA1B*, Liver hepatocellular carcinoma, Transcriptional signature, Signaling networks, Adrenergic receptors

## Abstract

The* α*_1A_-adrenergic receptor, encoded by *ADRA1A*, couples to heterotrimeric G_q/11_ proteins, promoting intracellular calcium mobilization and PKC activation. In human hepatocytes, it regulates acute metabolic responses and impacts gene expression. Although fundamental hepatic functions of *ADRA1A* have been described, its role in hepatocellular carcinoma remains elusive. We hypothesized that *ADRA1A* expressed in liver cancer tissue is part of a signaling repertoire potentially linked to patient outcomes. We analyzed The Cancer Genome Atlas liver cancer datasets and found *ADRA1A* gene deletion in 6% of patients and more prolonged survival of patients with high *ADRA1A* expression. Thus, we aimed to identify *ADRA1A*-signaling partners equally linked to more prolonged patient survival. Through a rational data mining strategy, based on the identification of the signaling repertoire linked to *ADRA1A* expression and statistical correlation with patient survival, we identified a transcriptional signature integrated by the adrenoceptor and five candidate signaling companions including two RhoGEFs: *FGD4* and *FARP2*, a phospholipid phosphatase: *PLPP6*, a RabGAP: *TBC1D2B*, and an RTK adaptor: *BAIAP2*. Additionally, highly co-expressed GPCRs such as *ACKR2*, *DRD1*, *ADRA1B*, *GPR17*, *S1PR1*, and *GPR182*, and RTKs like *KDR* and *TEK* similarly integrated transcriptional signatures with *ADRA1A* that correlated with more prolonged survival. Most *ADRA1A* candidate signaling partners were well-co-expressed with hepatocyte markers, suggesting that integrating *ADRA1A*-associated signaling networks could improve liver cancer patients’ prognosis.

**Supplementary Information:**

The online version contains supplementary material available at 10.1007/s40203-026-00706-4.

## Introduction

Hepatocyte proliferation occurring during liver regeneration is facilitated by α_1_-adrenergic receptors, which act as adjuvants of growth factors (Cruise et al. 1985; Cruise and Michalopoulos 1985; Michalopoulos and Bhushan 2021). In addition, α_1_-adrenergic agonists stimulate the differentiation of hepatoblasts from induced human pluripotent stem cells into albumin-positive hepatocyte-like cells (Kotaka et al. 2017). Mechanistically, all three α_1_-adrenergic receptors are coupled to the canonical phosphoinositide/calcium signaling pathway activated by the heterotrimeric G_q/11_ proteins (Garcia-Sainz et al. 1999; Vazquez-Prado and Garcia-Sainz 1996; Wu et al. 1992). However, the magnitude and duration of α_1_-adrenergic responses are characteristic of each subtype, indicating that differential expression levels, desensitization properties, and abilities of different α_1_-adrenergic receptor subtypes to activate non-canonical pathways likely impact their effects on acute and chronic physiological and pathological hepatic responses (Garcia-Sainz et al. 1999; Perez 2021). It is worth mentioning that the α_1_-adrenergic subtype expressed in hepatocytes varies among different species (Garcia-Sainz et al. 1992). Human hepatocytes express predominantly the α_1A_ subtype (Garcia-Sainz et al. 1995; Zhu et al. 2000).

α_1-_Adrenoceptors are known to transactivate growth factor receptors and the Src/Stat3 pathway (Han et al. 2008) and have been characterized as potential oncogenes (Allen et al. 1991). Whether proliferative mechanisms modulated by α_1_-adrenergic receptors play a role in hepatocellular carcinoma is an open question. In a preclinical model, in response to α_1A_-adrenergic receptor stimulation, hepatic stellate cells secrete soluble Frizzled-related protein 1 that activates the Wnt pathway and protumoral processes such as epithelial-mesenchymal transition, cell migration, and invasion in hepatocellular carcinoma cells (Lin et al. 2020). In contrast, two previous reports indicated that decreased expression of *ADRA1A* in hepatocellular carcinoma correlated with shorter patient survival (Chen et al. 2020; Zhao and Liu 2022), suggesting that *ADRA1A*-driven signaling networks might impact the progression of hepatocellular carcinoma, which represents the most frequent malignancy of the liver (Zhang et al. 2020). Decreased expression of *ADRA1A* was linked to hypermethylation of its promoter (Chen et al. 2020). In addition, *ADRA1A* transcript was targeted by miR-3682, which, by downregulating the adrenoceptor, impaired AMPK signaling, contributed to the malignant phenotype of hepatocellular carcinoma cells (Zhao and Liu 2022).

Moreover, *ADRA1A* knockdown promoted hepatocellular carcinoma cell proliferation, migration, and invasion (Zhao and Liu 2022), indicating a potential antitumoral role of *ADRA1A*-linked signaling pathways. Given the versatile role played by diverse GPCRs in cancer progression and the varied signaling repertoire available in cancer cells (Arang and Gutkind 2020; Li et al. 2023; O’Hayre et al. 2013), we postulate that α_1A_-adrenergic receptors might engage a particular set of candidate signaling partners having an impact on the progression of transformed cells, resulting in hepatocellular carcinoma. Revealing such putative impact of α_1_-adrenoceptor signaling on hepatic cancer will contribute to identifying biomarkers and designing effective targeted therapies. Here, through mining strategies, we surveyed the cancer landscape of hepatocellular carcinoma oncogenomic datasets focusing on screening the signaling repertoire associated with *ADRA1A* expression statistically linked to patient survival. We decoded *ADRA1A*-associated transcriptional signatures that correlated with more prolonged survival of liver cancer patients.

## Materials and methods

### *ADRA1A* expression and signaling landscape in TCGA pan-cancer studies

*ADRA1A* mRNA expression data of 31 TCGA cancer types (Sanchez-Vega et al. 2018), including hepatocellular carcinoma (LIHC), prostate adenocarcinoma (PRAD), low-grade glioma (LGG) and glioblastoma (GBM), among others, and transcriptomic data from paired normal liver tissue, were downloaded from the cBioPortal platform (https://www.cbioportal.org/) (Gao et al. 2013). Acute myeloid leukemia (LAML) did not contain *ADRA1A* mRNA data. Hepatocellular carcinoma genomic data was analyzed to identify *ADRA1A* gene copy number alterations and mutations. Similarly, gene expression of other α_1_-adrenergic receptors, their genetic alteration, and their coupled Gα subunits in 377 TCGA liver hepatocellular carcinoma patients were analyzed at the cBioPortal platform. Thus, two co-expression lists (90 patients per group, identified according to high or low *ADRA1A* expression), including all transcripts co-expressed with *ADRA1A*, were obtained for each group of patients. We focused on the higher correlated quartile, emphasizing those coding for signaling proteins, organized according to Spearman’s correlation coefficients. Signaling genes co-expressed with *ADRA1A* were identified from highly co-expressed transcripts organized according to their Spearman’s correlation values. Those with at least 0.2 Spearman’s value were initially selected. Additional selection criteria included higher correlation in patients with high versus low *ADRA1A* expression group, selecting those tagged as signaling partners. The labeling was based on the structural characteristics of the encoded proteins. *ADRA1A* candidate signaling companions were considered as those co-expressed transcripts with at least 0.2 Spearman’s correlation value, which was at least 0.05 higher in the high *ADRA1A* expression group and encoded for a signaling protein, which included receptors, kinases, phosphatases, G proteins, signaling adapters, among others, as defined by the SMART (http://smart.embl-heidelberg.de/) and Pfam (https://www.ebi.ac.uk/interpro/entry/pfam/#table) platforms. Additional selection was based on the comparative expression levels between normal and tumor tissues, choosing those *ADRA1A* candidate signaling partners with an expression pattern like *ADRA1A*. Comparative co-expression of *ADRA1A* candidate signaling partners with the two other α_1_-adrenergic receptors, *ADRA1B* and *ADRA1D*, was done using information from the respective co-expression datasets.

### Survival analysis

TCGA LIHC patients were divided according to *ADRA1A* expression. The OncoLnc platform (http://www.oncolnc.org/) was used to analyze the survival curves of selected patients within the 25:25 *ADRA1A* expression percentiles with 90 patients per group. Where indicated, survival curves were obtained from 50:50 percentiles with 182 patients in the low-risk group and 181 patients in the high-risk group. Integrated expression data of transcriptional signatures composed by *ADRA1A* and its candidate signaling companions were analyzed at the KM plotter platform (http://www.kmplot.com/analysis/index.php? p=service&cancer=custom_plot#) (Lanczky and Gyorffy 2021), looking for their statistical value as indicative of potential prognostic risk signatures linked to patient survival.

### Liver tumor cell markers

Co-expression analysis indicative of the cell composition within the LIHC tumor microenvironment, including stromal and cancer cells (Yin et al. 2018), was done by analyzing the Spearman’s correlation values of the selected transcripts with those of the following cell markers: *PCK1* for hepatocytes, *CD45* for immune cells, *ACTA2* for Ito cells (stellate), *PECAM1* for endothelial cells, *ITGAX* for dendritic cells and *CD68* for Kupffer cells. The list was obtained from the curated list of cell markers (Zhang et al. 2019), available at (http://xteam.xbio.top/CellMarker/).

### Enriched ***ADRA1A***-associated signaling pathways

The signaling repertoire associated to *ADRA1A* expression was analyzed for pathway enrichment and gene ontology at the Metascape platform (https://metascape.org/gp/index.html#/main/step1) (Zhou et al. 2019).

### Statistical analysis

T-tests and individual survival curves were made in GraphPad Prism 6.01. Transcriptional signatures’ accumulative risk was analyzed in KM plotter/Custom (http://www.kmplot.com/analysis/index.php?p=service&cancer=custom_plot#).

## Results

### High ***ADRA1A*** expression in hepatocellular carcinoma patients correlates with more prolonged survival

**Fig. 1:**
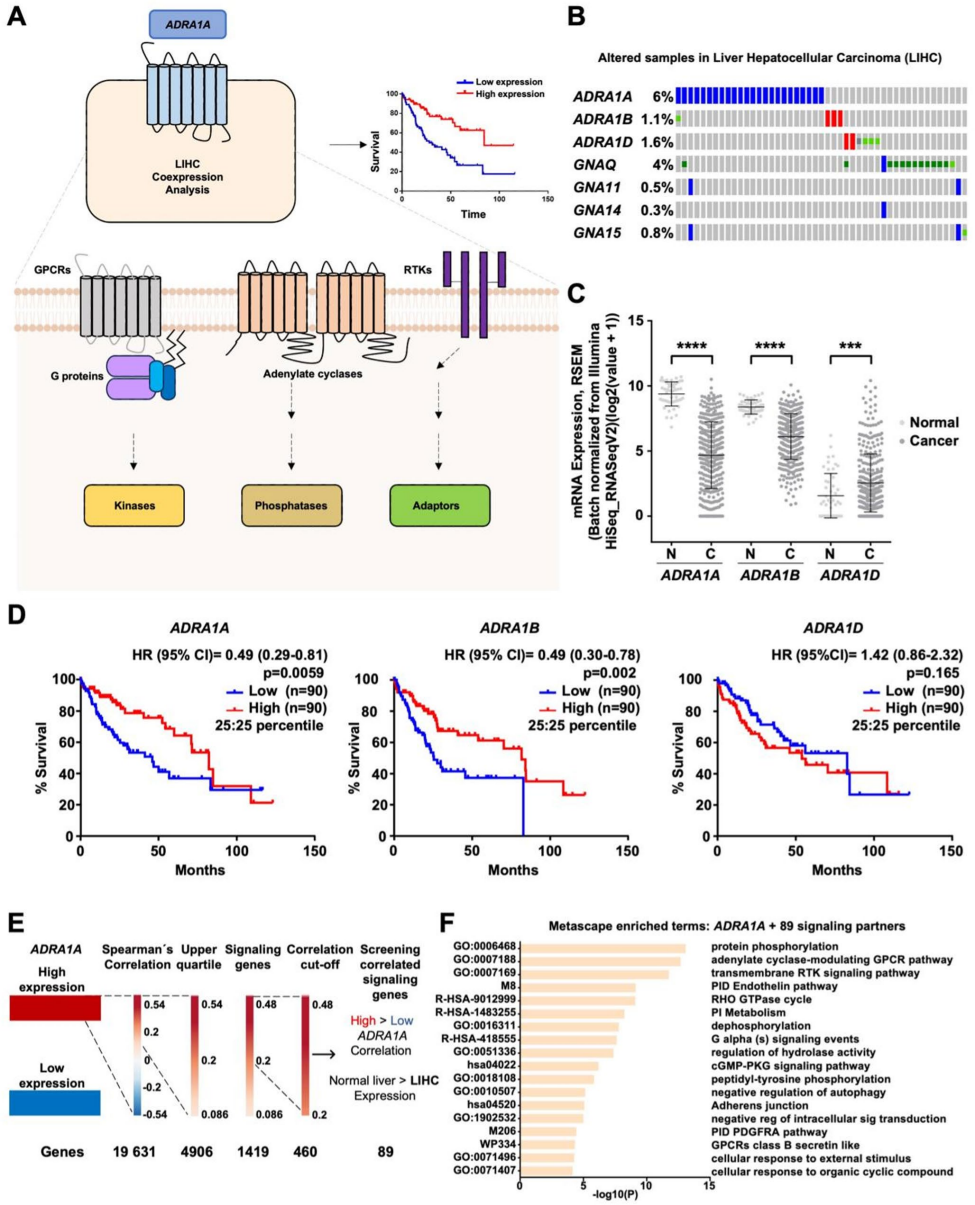
High expression of *ADRA1A* in hepatocellular carcinoma patients correlates with more prolonged survival. **A** Model depicting the hypothetical role of *ADRA1A* expression and signaling partners in liver cancer patient survival. **B** Oncoprint showing alteration frequency of adrenergic receptors and Gα_q/11_ proteins in TCGA Liver Hepatocellular Carcinoma (LIHC) patients analyzed at the cBioPortal platform. Deep deletion is indicated in blue, amplification in red, and point mutations with a green area in the middle of the grey symbol, each representing individual patients. Only patients with genetic alterations in the indicated genes are represented. **C** *ADRA1A*, *ADRA1B*, and *ADRA1D* mRNA expression in normal liver versus tumoral (cancer) tissue. ****p < 0.0001, ***p < 0.001 Unpaired t-test with Welch’s correction. Graphs represent the mean ± standard deviation. **D** Survival Kaplan-Meier analysis of LIHC patients based on *ADRA1A*, *ADRA1B*, and *ADRA1D* expression, comparing the higher and lower quartiles (90 patients per group). **E** Flowchart showing the mining strategy to identify *ADRA1A* candidate signaling partners. Guided by *ADRA1A* Spearman’s correlation coefficients with co-expressed transcripts in TCGA hepatocellular carcinoma datasets, the repertoire of co-expressed signaling partners was compared between two groups of patients, those at the top and bottom quartiles based on *ADRA1A* expression. **F** Enriched pathways identified at the Metascape platform with *ADRA1A* and its 89 candidate signaling partners identified according to the criteria represented in E

To identify the repertoire of G protein-coupled receptors, tyrosine kinase receptors, G proteins, kinases, phosphatases and adaptors, among other elements of the cellular signal transduction protein hardware, preferentially co-expressed with α_1A_-adrenergic receptors in cancer patients in which expression of this receptor correlated with patient survival (Fig. 1A), we initially compared *ADRA1A*’s mRNA expression in 31 cancer types from the TCGA datasets and analyzed whether expression of this receptor correlated with patient survival. Hepatocellular carcinoma (LIHC) was the second cancer type with the highest *ADRA1A* expression (Suppl. Figure 1). 6% of LIHC patients had a deep deletion of the *ADRA1A* gene (Fig. 1B). Genes encoding for the other α_1_-adrenoceptors (*ADRA1B* and *ADRA1D*) exhibited less than 2% alterations. In the case of the Gα subunits of the family of heterotrimeric G proteins known to be coupled to these receptors, *GNAQ* (encoding for Gα_q_) was the most frequently altered, found mutated in 4% LIHC patients (Fig. 1B). The most frequent mutation *GNAQ* T96S, found in this group, has been characterized as having oncogenic properties in liver cancer cells (Choi et al. 2021). The *ADRA1A* transcript was less abundant in LIHC tumors compared to the normal liver (Fig. 1C), as was the case for *ADRA1B* but not *ADRA1D* (Fig. 1C). Higher *ADRA1A* expression in hepatocellular carcinoma patients was statistically correlated with more prolonged survival (Fig. 1D, left panel). A similar statistically significant difference was observed for patients with high *ADRA1B* (Fig. 1D, middle panel) but not *ADRA1D* (Fig. 1D, right panel), raising the possibility that α_1A_- and α_1B_-adrenergic receptors are linked to signaling pathways that somehow attenuate cancer progression in liver hepatocarcinoma patients. Given that *ADRA1A* was deleted in 6% of patients and exhibited significantly less expression in tumors than in normal liver tissue, we focused on identifying the signaling repertoire associated to *ADRA1A* expression and patient survival.

Aiming to discover *ADRA1A*-associated transcriptional signatures statistically correlated to patient survival, we analyzed the TCGA LIHC transcriptional datasets looking for *ADRA1A* co-expressed transcripts. We divided the transcriptional profile co-expressed with *ADRA1A* into two subgroups of patients: those with high and low *ADRA1A* expression. As depicted in Fig. 1E, 19,631 transcripts had a Spearman’s correlation value indicative of its level of co-expression with *ADRA1A*. We selected the upper quartile from both groups of patients to focus on positively correlated genes. We then chose those coding for signaling proteins and selected those with at least 0.2 Spearman’s correlation coefficient.

With these criteria, the group was reduced to 460 signaling genes. Subsequently, we selected those with higher Spearman’s correlation value in the group of patients with high *ADRA1A* expression and which, similarly to *ADRA1A*, were less abundant in tumors compared to normal liver tissue, resulting in a group of 89 *ADRA1A* signaling companions (Fig. 1E). To address the potential functionality of that signaling repertoire, we analyzed the data at the Metascape platform for enriched signaling pathways. *ADRA1A* and its candidate signaling partners were predictably linked to various GPCR-regulated molecular pathways (Fig. 1F).

**Fig. 2:**
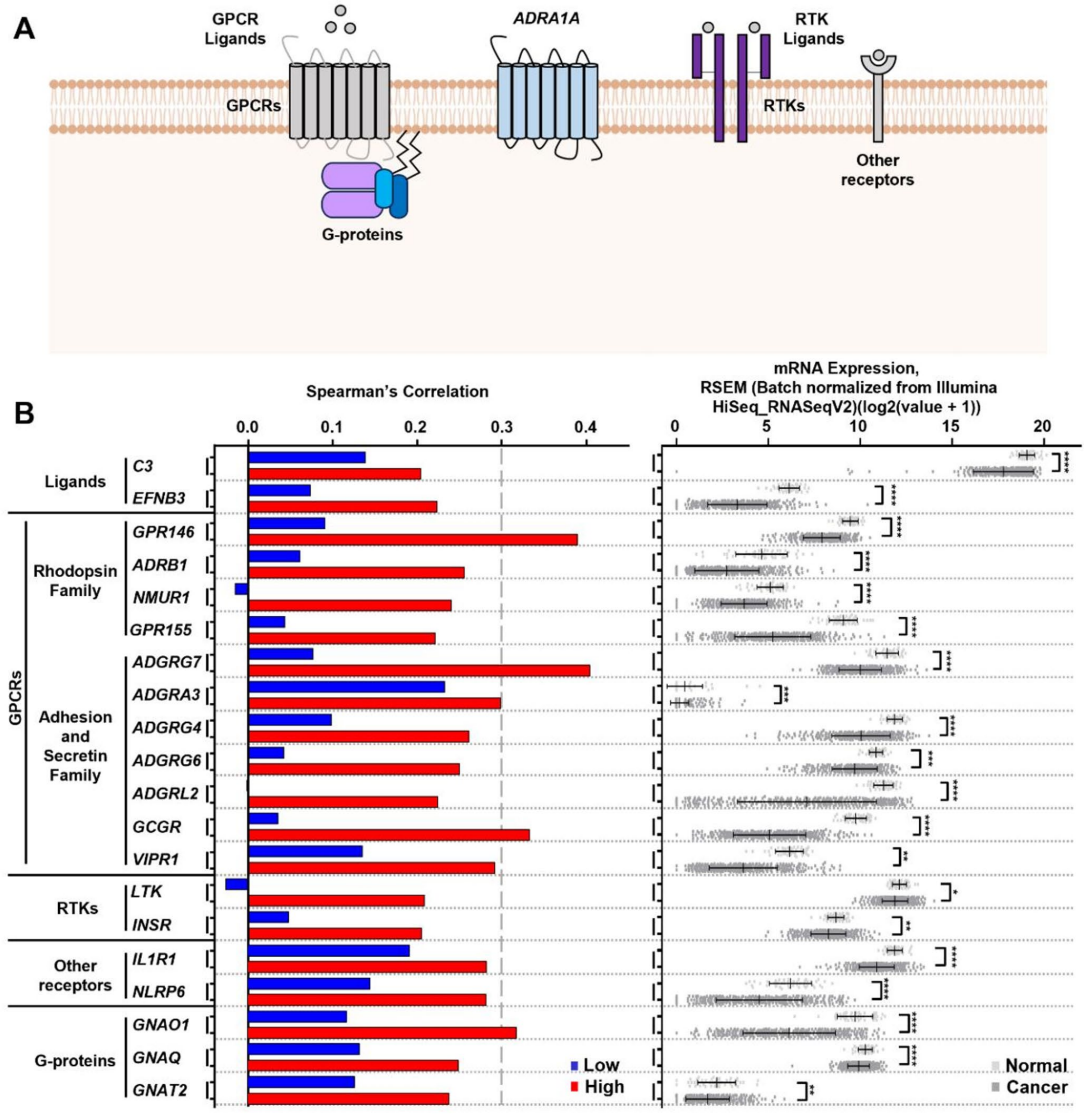
Ligands, receptors, and G-proteins highly co-expressed with *ADRA1A* in hepatocellular carcinoma patients. **A** Model depicting peptidic ligands, receptors, and heterotrimeric G proteins co-expressed genes with *ADRA1A* in LIHC. **B** Ligands, receptors, and G-proteins genes highly co-expressed with *ADRA1A* in the group of patients with high *ADRA1A* expression. All with significantly higher expression in normal versus cancer liver tissues. Data represented in the graph at the left correspond to Spearman’s correlation values of the indicated transcripts co-expressed with *ADRA1A*. The graph at the right shows their mRNA expression in normal and cancer liver tissues. Means ± standard deviations are indicated. ****p < 0.0001, ***p < 0.001, **p < 0.01, *p < 0.05, Unpaired t-test with Welch’s correction

**Fig. 3:**
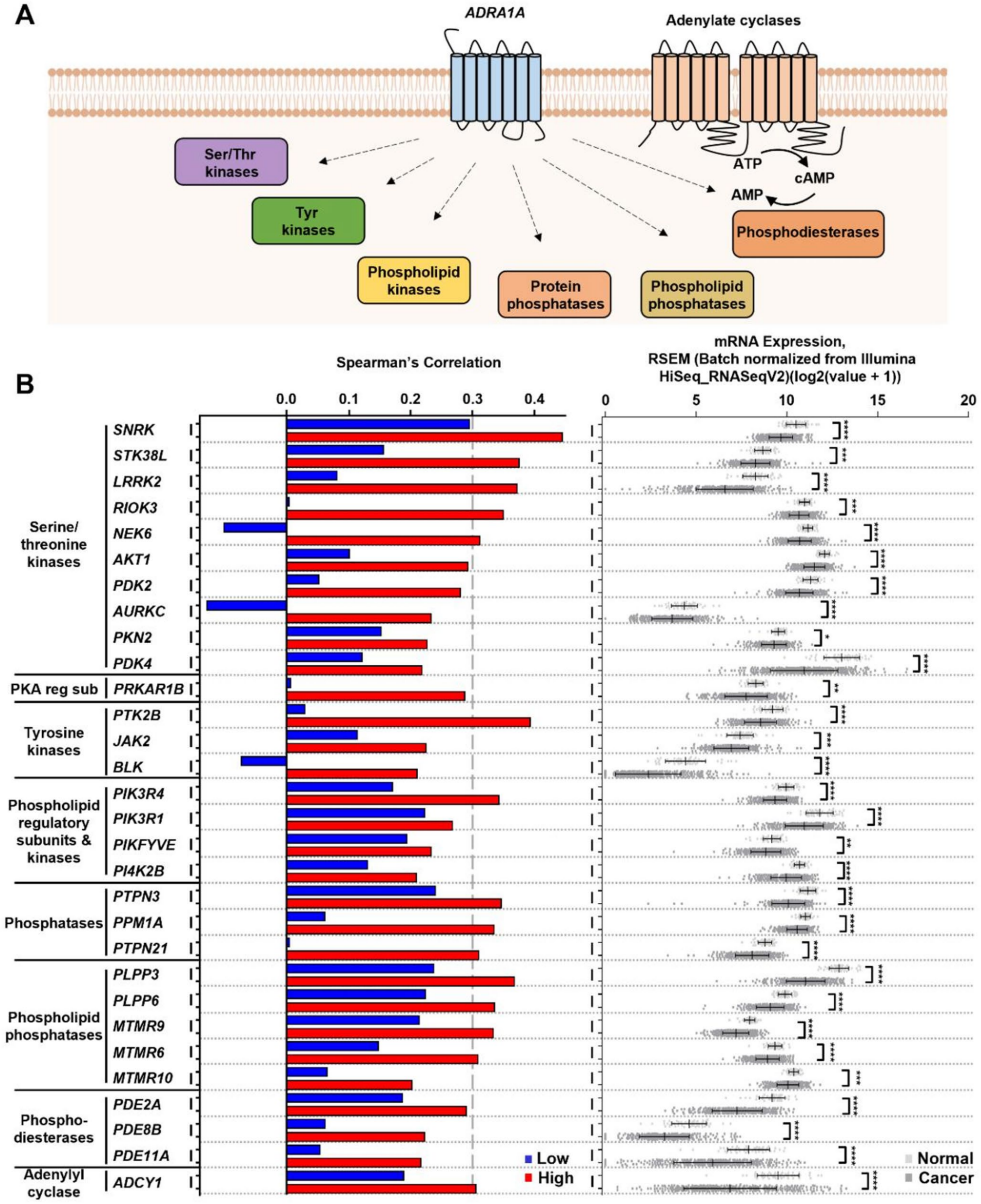
Kinases, phosphatases, and regulators of cAMP production and degradation highly co-expressed with *ADRA1A* in hepatocellular carcinoma patients. **A** Model representing kinases and phosphatases of different specificities, adenylate cyclases, and phosphodiesterases correlated with *ADRA1A*. **B** Candidate signaling partners of *ADRA1A* involved in protein and lipid phosphorylation and dephosphorylation, and cAMP production and degradation highly co-expressed with *ADRA1A* in patients divided by low and high *ADRA1A* expression (left). The graph on the right represents their mRNA expression in normal and cancer liver tissues. Means ± standard deviations are represented. ****p < 0.0001, ***p < 0.001, **p < 0.01, *p < 0.05, Unpaired t-test with Welch’s correction

**Fig. 4:**
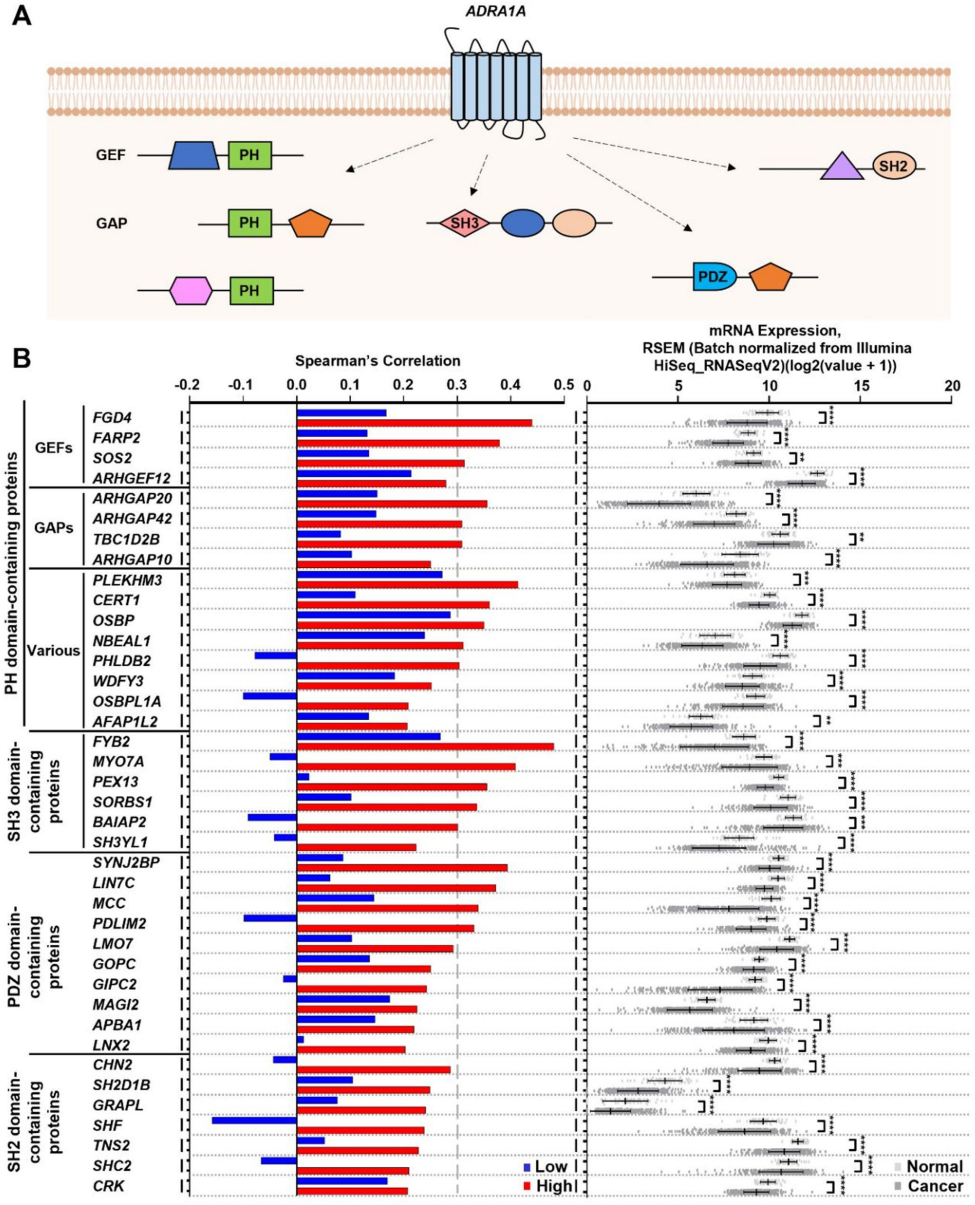
*ADRA1A* candidate signaling partners containing PH, SH3, PDZ, or SH2, interaction domains highly co-expressed in hepatocellular carcinoma patients. **A** Model of *ADRA1A* candidate signaling partners having characteristic interaction domains. **B** Co-expressed transcripts coding for proteins with PH, SH3, PDZ, and SH2 interaction domains are represented according to their Spearman’s co-expression values with *ADRA1A* in the low- and high-*ADRA1A* expression groups (left). Their mRNA expression in normal versus cancer liver tissues is shown at the graph on the right. Graph represents individual values and their corresponding mean ± standard deviation. ****p < 0.0001, ***p < 0.001, **p < 0.01, Unpaired t-test with Welch’s correction

Given that α_1_-adrenergic receptors are known to be cross-regulated by diverse co-expressed receptors, a situation that has been mainly documented in the case of α_1B_-adrenoceptors (Casas-Gonzalez and Garcia-Sainz 2006; Casas-Gonzalez et al. 2000, 2003; Castillo-Badillo et al. 2012; del Carmen Medina et al. 2000; Garcia-Sainz et al. 2004; Gonzalez-Arenas et al. 2006; Medina et al. 1998; Molina-Munoz et al. 2008; Romero-Avila et al. 2002; Vazquez-Prado et al. 1997, 2003), we wanted to get an initial insight into the signaling landscape present in the context of high *ADRA1A* expression in LIHC patients, also coinciding with *ADRA1A* in terms of exhibiting significantly less expression in tumors compared with normal liver tissue. We identified a group of ligands, receptors, and diverse members of the intracellular signaling hardware preferentially co-expressed with *ADRA1A* in LIHC tumors with high expression of this receptor and less abundant in tumors compared to normal liver tissue. Figures two to four present the *ADRA1A* candidate signaling partners as they work in a prototypical cascade. Figure 2 shows peptide ligands, receptors, and G protein α subunits; Fig. 3 shows kinases, phosphatases, and other signaling effectors related to protein phosphorylation and second messenger production and degradation; and Fig. 4 shows signaling effectors and adaptors containing interaction domains such as PH, SH2, SH3, and PDZ.

### Ligands, receptors, and G-proteins highly co-expressed with ***ADRA1A*** in hepatocellular carcinoma patients

The transcripts coding for protein/peptide ligands preferentially co-expressed with *ADRA1A* in TCGA LIHC patients with high adrenoceptor expression (Fig. 2A) and less expressed in tumors than normal liver tissue, included *C3*, and *EFNB3*. Regarding receptors, eleven GPCRs, including four from the rhodopsin family (*GPR146*, *ADRB1*, *NMUR1*, *GPR155*) and seven from the adhesion and secretin families (*ADGRG7*, *GCGR*, *ADGRA3*, *VIPR1*, *ADGRG4*, *ADGRG6*, *ADGRL2*), two receptor tyrosine kinases (*LTK*, *INSR*), two non-GPCR/RTK receptors (*IL1R1*, *NLRP6*) and three Gα subunits of heterotrimeric G proteins (*GNAO1*, *GNAQ*, *GNAT2*) were preferentially co-expressed with *ADRA1A* in the high expression group. All these co-expressed genes had Spearman’s correlation values of at least 0.2 with *ADRA1A*, preferentially in the high *ADRA1A* expression group (Fig. 2B, red bars), and were less expressed in liver tumors compared to normal liver tissue (Fig. 2B, right), which, as shown in Fig. 1C, was the case for *ADRA1A*.

### Kinases, phosphatases, and effectors linked to second messenger production and degradation highly co-expressed with ***ADRA1A*** in hepatocellular carcinoma patients

Continuing with the identification of potential signaling partners of *ADRA1A*, the following categories were kinases, phosphatases, and effectors such as adenylyl cyclases, phosphodiesterases, and phospholipid phosphatases, involved in second messenger production or degradation (Fig. 3A). We identified transcripts from ten serine/threonine kinases, one PKA regulatory subunit, three cytosolic tyrosine kinases, two phospholipid kinase regulatory subunits, two phospholipid kinases, one phospho-serine/threonine phosphatase, two phospho-tyrosine phosphatases and five phospholipid phosphatases. In addition, we identified transcripts from three phosphodiesterases and one adenylyl cyclase (Fig. 3B). All of them exhibited a Spearman’s correlation value of at least 0.2 with *ADRA1A* in the high expression group and had lower mRNA levels in cancer than normal tissues (Fig. 3B, right).

### ***ADRA1A*** signaling companions with PH, SH2, SH3, and PDZ interaction domains in hepatocellular carcinoma patients

Considering the relevance of small G proteins and protein-protein interactions in cancer progression, we wanted to identify the group of small G protein regulators and signaling proteins with interaction domains that were preferentially co-expressed with *ADRA1A* in the group of LIHC patients with high expression of the adrenoceptor. We focused on guanine nucleotide exchange factors (GEFs) and GTPase activating proteins (GAPs), among other effectors and adapters characterized by the presence of PH, SH2, SH3 and PDZ domains (Fig. 4A). Among the transcripts coding for signaling proteins that contain PH domains we found four GEFs, including *FGD4* and *FARP2* as those with highest Spearman’s correlation values and four GAPs, from which *ARHGAP20* was the best co-expressed (Fig. 4B). None of these regulators of small GTPases have been previously studied in the context of *ADRA1A* signaling. In addition, we identified six *ADRA1A* co-expressed transcripts coding for proteins with SH3 domains, ten with PDZ domains, and seven with SH2 domains (Fig. 4B), all of them with an expression profile like *ADRA1A*, lower in cancer compared with normal tissue (Fig. 4B, right panel).

### Signaling genes co-expressed with ***ADRA1A*** correlate with hepatocyte markers and more prolonged survival of LIHC patients

**Fig. 5:**
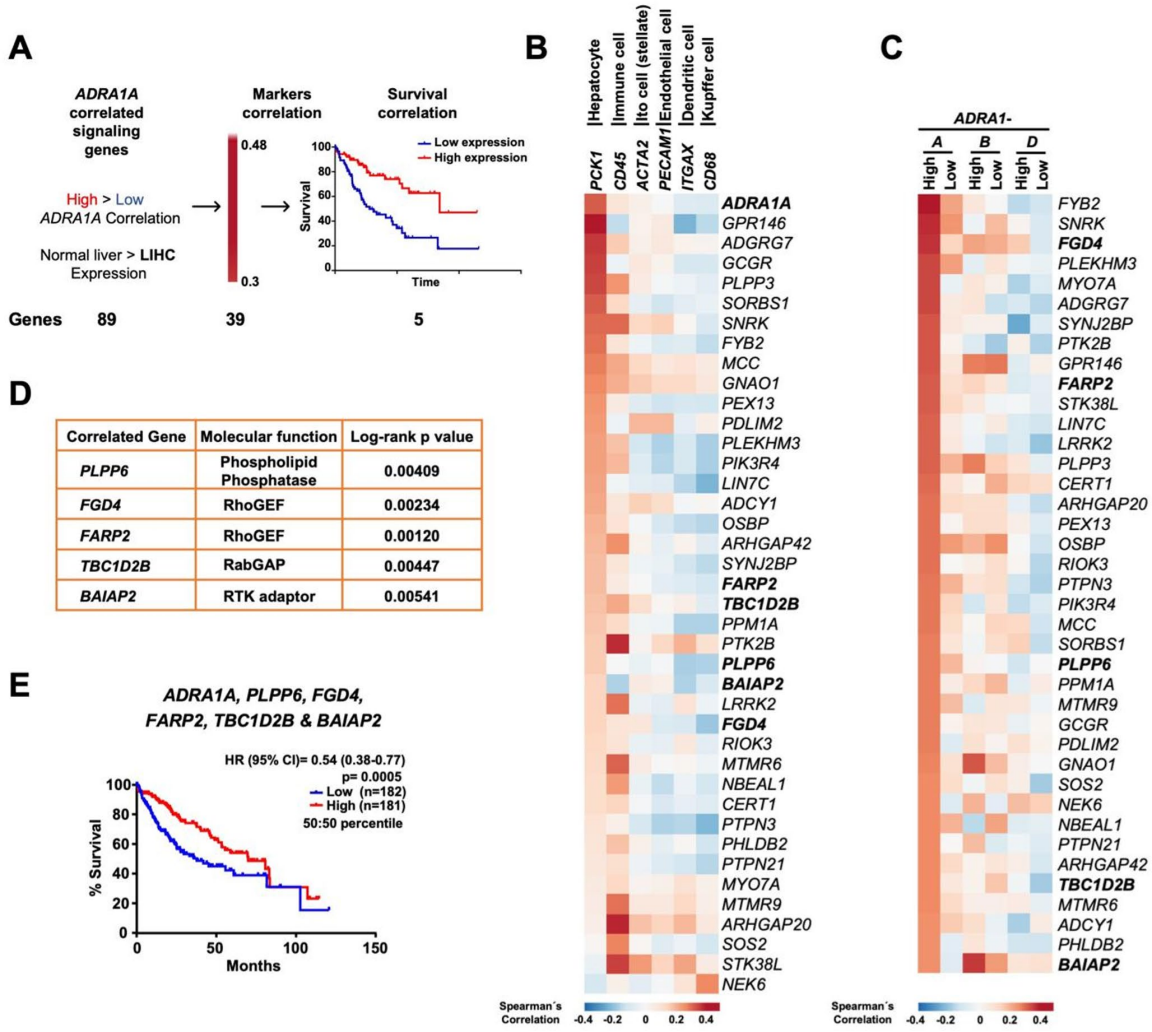
*ADRA1A* co-expressed signaling genes correlate with more prolonged survival of LIHC patients. **A** *ADRA1A* candidate signaling partners, identified as shown in Figs. 2, 3 and 4, were further screened to select those with Spearman’s correlation values with *ADRA1A* of at least 0.3. Each was then analyzed regarding expression and patient survival to select those whose higher expression correlated with more prolonged survival. **B** Heatmap showing the co-expression analysis of *ADRA1A* candidate signaling partners with markers of hepatocytes and tumor stroma cells. Higher correlation values are represented by darker tones of red and negative correlation values are represented in blue. **C** Heatmap representing the Spearman’s correlation values of *ADRA1A* candidate signaling partners with different α_1_-adrenergic receptors divided in high and low expression groups. **D** *ADRA1A* candidate signaling partners whose high expression correlated with more prolonged patient survival. Survival was analyzed in high and low quartiles (90 patients per group). Log-rank p values were obtained from the OncoLnc platform. **E** Overall survival of TCGA LIHC patients based on the risk score defined by the *ADRA1A*-associated transcriptional signature composed by *ADRA1A* and its indicated candidate partners of the intracellular signaling hardware. The survival curve was obtained by Univariate Cox regression analysis in KM plotter with patients split by median expression

From the 89 candidate signaling genes that correlated with *ADRA1A* in the high expression group of LIHC patients and were less expressed in tumors compared to normal liver tissues (similar to *ADRA1A* expression profile), we selected those with higher Spearman’s correlation values (Fig. 5A, 0.3 Spearman’s coefficient, shown as bars crossing the dotted lines in Figs. 2B and 3B, and 4B). Given that the highest Spearman coefficient in LIHC screened dataset was 0.48, we decided to apply a higher cut-off value to highlight those co-expressed signaling genes with better possibilities to be linked in a common pathway. This cutoff resulted in a group of 39 candidate signaling companions of *ADRA1A* (three GPCRs, one G-protein, one adenylyl cyclase, seven kinases, seven phosphatases, eleven proteins with PH domains, five with SH3 domains, and four with PDZ domains) which were compared in their co-expression with cell markers that represent different cell populations within the liver tumors, from hepatocytes to stromal and immune cells (Fig. 5B). *ADRA1A* and most of its candidate signaling companions correlated with the hepatocyte marker (*PCK1*), suggesting that the identified *ADRA1A*-associated transcriptional signature is relevant in cancerous liver cells (Fig. 5B). Next, we tested whether these candidate signaling companions were preferentially co-expressed with *ADRA1A* in the high expression group, compared to the other α_1_-adrenoceptors, *ADRA1B* and *ADRA1D*. *ADRA1A*, which guided the identification of these candidate signaling genes, was indeed the α_1_-adrenoceptor preferentially co-expressed with the different members of the 39 signaling companions, showing the highest Spearman’s correlation values in the high *ADRA1A* expression group. Most of these candidate signaling genes were less co-expressed with *ADRA1B* (except *GNAO1* and *BAIAP2*, which had higher co-expression with *ADRA1B* than *ADRA1A*), and negative co-expression values were detected with *ADRA1D* (Fig. 5C).

We then wanted to know which of these 39 candidate signaling genes correlated statistically with patient survival. We further focused our analysis on those whose high expression correlated with prolonged survival, like *ADRA1A*, to consolidate an associated transcriptional signature. This *ADRA1A*-associated signature could be linked, at least in terms of higher statistical correlation, with improved potential value compared to a single expressed transcript. We found that high expression of five candidates, *PLPP6*, *FGD4*, *FARP2*, *TBC1D2B*, and *BAIAP2*, correlated with more prolonged survival of LIHC patients (Fig. 5D). We then evaluated them all together as a transcriptional signature potentially indicative of differential risk among LIHC patients. We found that *ADRA1A*-associated transcriptional signature significantly correlated with lower risk for LIHC patients (Fig. 5E), indicating the possibility that as an integrated signaling system, *ADRA1A* and these candidate signaling companions regulate hepatocyte pathways that somehow attenuate cancer progression in patients with liver cancer.

### GPCRs and RTKs co-expressed with ***ADRA1A*** correlate with more prolonged survival of LIHC patients

**Fig. 6:**
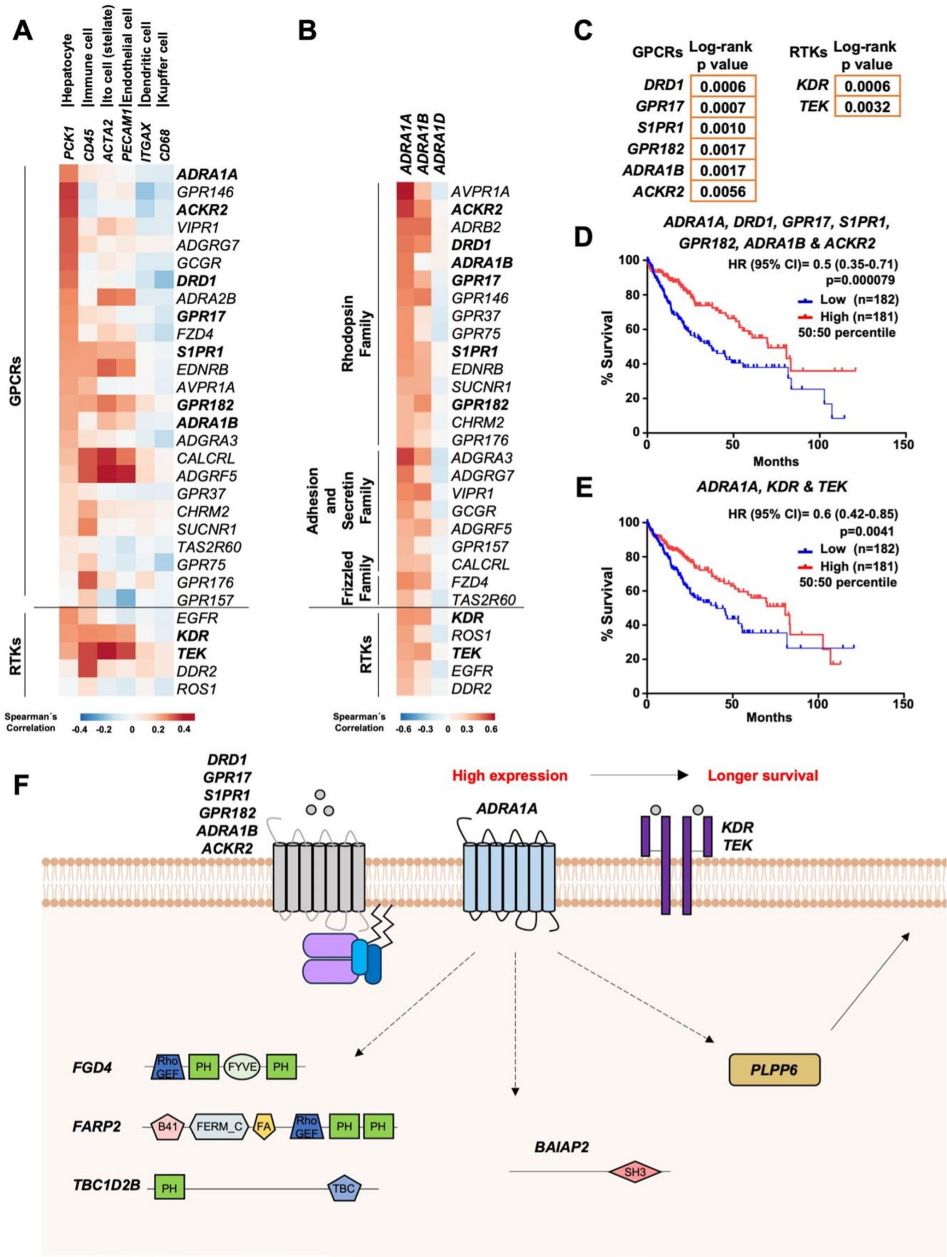
*ADRA1A* co-expressed GPCRs and RTKs correlate in hepatocytes and with more prolonged survival. **A** Heatmap representing the Spearman’s correlation values of GPCRs and RTKs, identified as *ADRA1A* candidate signaling partners, with markers of hepatocytes and stromal cells in cancer tissues of TCGA LIHC patients. The highest correlations are indicated in darker tones of red as shown in the bar at the bottom. **B** Comparative analysis of GPCRs and RTKs co-expression with the three α_1_-adrenergic receptors transcripts. GPCRs and RTKs transcripts were identified by their co-expression with *ADRA1A* in all TCGA LIHC patients selected by their Spearman’s correlation values of at least 0.3. The bar at the bottom of the heatmap indicates negative to positive correlations. **C** GPCRs and RTKs whose high expression correlated with more prolonged survival. The indicated p values were obtained from individual survival curves analyzed at the OncoLnc platform. Patients were segregated into quartiles based on the expression of the indicated transcripts and the top and bottom groups were analyzed (90 patients per group). **D** *ADRA1A*-associated GPCRs signature correlation with patient survival. **E** *ADRA1A*-associated RTKs signature correlation with patient survival. **D-E**, Overall survival of TCGA LIHC patients split by median based on the risk score defined by the indicated *ADRA1A*-associated transcriptional signatures. Univariate Cox regression analysis at the KM plotter platform resulted in the depicted survival curves. **F** Model showing *ADRA1A* and its candidate signaling companions, included in *ADRA1A*-associated transcriptional signaling signatures, statistically correlated with lower risk for patients with LIHC

Considering the intertwined regulation among GPCRs and growth factor receptors (Vazquez-Prado et al. 2003), we searched for the identity of the receptors of these main groups that were highly co-expressed with *ADRA1A*. We selected those transcripts coding for receptors with at least 0.3 Spearman’s correlation coefficient in the list of co-expressed transcripts that included all LIHC patients and analyzed their co-expression with cell markers, as done with the candidate signaling companions shown in Fig. 5B. The GPCRs co-expressed with *ADRA1A* included *ACKR2*, *DRD1*, and *GPR17*, that had the highest Spearman’s correlation values with *PCK1*, the hepatocyte marker (Fig. 6A). Others, including *S1PR1*, *GPR182* and *ADRA1B* were co-expressed not only with the hepatocyte marker but also with markers of other cells, including immune, stellate and endothelial cells, as in the cases of *S1PR1* and *GPR182* (Fig. 6A). Regarding *ADRA1B*, the transcript of this receptor was co-expressed with hepatocyte, stellate and, at lower degree, endothelial markers. From the group of five transcripts coding for RTKs identified as *ADRA1A* candidate signaling partners co-expressed with the hepatocyte marker (Fig. 6A), *KDR* and *TEK* were also co-expressed with markers of other cells of the tumor stroma, including immune, stellate, and endothelial cells (Fig. 6A, bottom part). When the co-expression of these GPCRs and RTKs with *ADRA1A* was compared with their co-expression with other adrenoceptors of the family, most of them were well co-expressed with both *ADRA1A* and *ADRA1B*, but not with *ADRA1D* (Fig. 6B). Among the group of GPCRs and RTKs co-expressed with *ADRA1A*, the high expression of six transcripts coding for GPCRs (*DRD1*, *GPR17*, *S1PR1*, *GPR182*, *ADRA1B* and *ACKR2*) and two transcripts coding for RTKs (*KDR* and *TEK*) was significantly correlated with more prolonged patient survival (Fig. 6C), as it was the case for *ADRA1A* (Fig. 1D, left panel). Thus, we tested whether *ADRA1A* with six GPCRs or two RTKs candidates (indicated in Fig. 6C) were statistically correlated with patient survival when analyzed as signaling signatures. Consistent with a potentially reduced risk for LIHC patients, *ADRA1A* + 6 GPCRs (Fig. 6D) and *ADRA1A* + 2 RTKs associated transcriptional signatures (Fig. 6E) were statistically correlated with more prolonged patient survival.

## Discussion

The multiomic approach to study cancer has enriched our view to a more comprehensive understanding of altered molecular pathways, guiding the characterization of druggable targets and prognostic biomarkers (Li et al. 2023; Network 2017; Smith and Sheltzer 2022). Here, we analyzed the TCGA LIHC transcriptomic datasets, looking for co-expressed transcripts coding for candidate signaling partners of the α_1A_-adrenergic receptor statistically correlated with more prolonged patient survival. We identified three *ADRA1A*-associated transcriptional signaling signatures. Their components are depicted in Fig. 6F as pieces of putative signaling hardware potentially linked to better prognosis of LIHC patients. The first is integrated by *ADRA1A* and five candidate components of the intracellular signaling hardware, including two RhoGEFs and one RabGAP. The second and third signatures included candidate GPCRs and RTKs linked to *ADRA1A* expression (Fig. 6F).

Most components of these three *ADRA1A*-associated signaling signatures have never been studied in the context of* α*_1A_-adrenergic receptor signaling, providing support to future investigations on the α_1A_-adrenergic pathways in liver hepatocellular carcinoma, based on the potential benefit of activating these pathways in LIHC patients. Our analysis, based on transcriptomic co-expression and statistical significance of patient survival, raises the question of how, given the case, *ADRA1A*-associated signaling signatures would affect tumor biology resulting in extended patient survival. Although no mechanistic conclusions can be drawn from our analysis, our emerging hypothesis postulate that these *ADRA1A*-associated signaling signatures could impact communication among cells within the tumor microenvironment, from liver cell-autonomous to paracrine communication with immune cells (*TBC1D2B* in signature 1) and stellate cells (*GPR182* in signature 2). In addition, a regulatory role on endothelial cells is predicted, based on the presence of *S1PR1* and *GPR182* in signature 2, and *KDR* and *TEK* in signature 3, in this regard, the effect of S1PR on blood vessel normalization (Cartier et al. 2020; Weigel et al. 2023) and its ability to control immune cell migration (Choi and Jung 2023; Hallisey and Schwab 2023) are consistent with this possibility. Interestingly, communication between S1PR1 and adrenergic receptors has been established at the molecular level (Castillo-Badillo et al. 2012). Although our systematic data mining approach was focused on the pieces of the signaling repertoire that are linked by expression to *ADRA1A*, and statistically correlated with more prolonged patient survival, a functional biochemical pathway remains to be investigated. It is worth mentioning that these *ADRA1A*-associated transcriptional signatures should get validated with an independent liver cancer database like studies in the GEO repository, ICGC LIRI-JP or HCCDB databases to confirm their robustness.

The α_1A_-adrenergic receptor activates liver metabolism and participates in proliferation and regeneration (Goodhardt et al. 1984; Han et al. 2008; Michalopoulos and DeFrances 2005; Schmelck and Hanoune 1980). Here we found it as a putative pro-survival receptor in liver cancer. Patients with high expression of *ADRA1A* exhibited more prolonged survival, which suggests that the α_1A_-adrenergic receptor promotes the “normalization” of signaling pathways, leading to better patient prognosis. We then investigated the identity of *ADRA1A* candidate signaling partners statistically linked to more prolonged patient survival. They included transcripts from protein and lipid kinases and phosphatases, GPCRs, RTKs, RhoGEFs, and RhoGAPs (Figs. 2B and 3B, and 4B).

*FGD4* and *FARP2* were the candidate RhoGEFs identified in the first transcriptional signature, possibly acting as* α*_1A_-adrenergic receptor intermediate effectors. FGD4 is a Cdc42-GEF with an actin-binding domain (ABD) that serves as a crosslinker of actin filaments (Bossan et al. 2018). FARP2 is a Rac/Cdc42-GEF that contributes to cell-cell adhesions (Elbediwy et al. 2019). We speculate that its potential function in the α_1A_-adrenergic receptor pathway is consistent with its property of promoting and keeping cell adhesions and maintaining cell polarity, which is considered a mechanism of tissue normalization by preventing mesenchymal transition, typical of advanced cancer cells. Given that α_1A_-adrenergic receptor is known to activate PKC, this kinase might constitute a common regulatory node by which the group of candidate signaling partners, identified by co-expression with the receptor, could be integrated in a signaling network linked to a better outcome of hepatic cancer patients. Consistent with this possibility, FGD4 has been identified as a PKCα partner in the Bioplex 3.0 interactome dataset (Huttlin et al. 2021). In addition, several potential phosphorylation sites by PKC are recognizable by Scansite analysis in FGD4, FARP2, PLPP6 and TBC1D2B, a possibility that warrants experimental confirmation. While FGD4 has been found overexpressed in prostate cancer promoting proliferation and migration (Bossan et al. 2018), *FGD4* deletions correlated with shorter survival of hepatoblastoma patients (Udomwimonsit et al. 2026), which is consistent with a pro-survival role of this protein in liver cancer. The phospholipid phosphatase 6 (PLPP6) catalyzes the dephosphorylation of phosphatidic acid, sphingosine 1-phosphate, lysophosphatidic acid and ceramide 1-phosphate (Miriyala et al. 2010; Theofilopoulos et al. 2008). As an α_1A_-adrenergic receptor candidate companion, it could regulate phospholipid receptors, like S1PR and LPAR, through the dephosphorylation and availability of their ligands. The RabGAP TBC1D2B could be involved in the control of α_1A_-adrenergic receptor intracellular trafficking, known to involve Rab GTPases which, depending on the agonist, can direct the receptor to early or late endosomes with the participation of Rab5 and Rab7 GTPases, respectively (de-Los-Santos-Cocotle et al. 2020; Martinez-Morales et al. 2022). The co-expression between *BAIAP2*, coding for the insulin receptor substrate IRSp53, and *ADRA1A* is consistent with the existence of signaling crosstalk as it has been documented for* α*_1B_-adrenergic receptor, and various RTKs, including those for Insulin, EGF, and PDGF (Garcia-Sainz et al. 2000; Vazquez-Prado et al. 2003). The identification of *BAIAP2* as part of a pro-survival signature is consistent with the reported contrasting effect of a non-coding antisense RNA complementary to *BAIAP2*, BAIAP2-AS1, which promotes hepatocellular carcinoma (Yang et al. 2021), supporting the idea that high expression of *BAIAP2* could translate to IRSp53 somehow collaborating with the α_1A_-adrenergic receptor to attenuate liver cancer progression. However, the effect seems to be dependent on the oncogenic context, since pro-invasive phenotypes have been linked to *BAIAP2* expression in other cancer types, like bladder and prostate cancer (Huang et al. 2025; Quan et al. 2022).

Regarding the potential communication between the α_1A_-adrenergic receptor and other receptors, we found six GPCRs and two RTKs as candidates highly correlated with *ADRA1A* and with more prolonged patient survival (Fig. 6D-E). Each associated signature had higher statistical significance than *ADRA1A* alone. Although the experimental demonstration of the hypothetical consequences of receptor crosstalk suggested by our analysis is beyond our current data-mining strategy, we postulate that *ACKR2*, *DRD1*, and *GPR17* could be part of cell-autonomous crossed-communication pathways that contribute to delay liver tumor progression. In the case of *ADRA1B*, *S1PR1*, and *GPR182*, they correlate with tumor stromal cell markers, suggesting the integration of cell-autonomous mechanisms with paracrine communication with cells of the tumor stroma. Co-expression of *ADRA1A* and *ADRA1B* is particularly interesting because they respond to the same endogenous agonists. However, the α_1A_-adrenergic receptor might be a more precise therapeutic target of subtype-selective ligands because *ADRA1A* correlated exclusively in hepatocytes. In the case of the dopamine D1 receptor (*DRD1*), its ability to regulate α_1A_-adrenergic receptor expression has been documented in renal proximal tubule cells (Ennis et al. 2014). Dopamine D1 receptor regulates α_1A_-adrenergic receptor localization and signaling at the plasma membrane (Ennis et al. 2014). GPR182 is expressed in liver sinusoidal endothelial cells (Le Mercier et al. 2021; Schmid et al. 2018) and is known to undergo phenotypic changes due to hepatic carcinoma, which diminishes the immune response against the tumors (Gracia-Sancho et al. 2021). We speculate that the potential crosstalk between GPR182 and* α*_1A_-adrenergic receptors could contribute to optimal signaling pathways against the alteration of endothelial cells. *ADRA1A* correlated with two endothelial RTKs at the transcriptional level, *KDR* and *TEK* coding for vascular endothelial growth factor receptor 2 and the angiopoietin-1 receptor, respectively, pointing to a communication mechanism between cancerous and stromal cells. Such type of paracrine communication has been documented in the heart, where cardiomyocytes overexpressing α_1A_-adrenergic receptors upregulate VEGF-A, which activates endothelial cells to promote an angiogenic response, relevant in a post-infarct transgenic rat model (Zhao et al. 2015). The correlation of the identified GPCRs and RTKs and α_1_-adrenergic receptors was higher with *ADRA1A* than with other α_1_-adrenergic receptors (Fig. 6B). However, *ADRA1B* was also well co-expressed with various *ADRA1A* signaling partners, suggesting high parallelism in the role for α_1A_- and α_1B_-adrenergic receptors in hepatocytes, but distinct from α_1D_-adrenergic receptors, which seem to be the most functionally distinct among the subtype family (Rodriguez-Perez et al. 2009).

The three identified *ADRA1A*-associated signaling signatures correlated with phosphoenolpyruvate carboxykinase (*PCK1*), a hepatocyte marker (Aizarani et al. 2019; MacParland et al. 2018), suggesting the existence of hepatocyte-autonomous mechanism by which α_1A_-adrenergic receptor could attenuate tumor progression in LIHC patients. However, some elements might involve communication with immune and endothelial cells (Fig. 5B). Here, we identified signaling signatures potentially helpful to stratify patients for a better outcome. Similarly, in hepatocellular carcinoma, adenylosuccinate lyase (ADSL) has been validated as a pan-cancer prognostic and immune biomarker (Zhu et al. 2025a, b), an aspartate metabolism-related gene signature (AMGS) has been defined as a set of promising biomarkers (Shi et al. 2024), as has been mutational signatures that indicate tumor immunogenicity (Zhou et al. 2025). A contrasting feature falls in the hepatocyte-specific signature integrated by *ADRA1A* versus ADSL as a pan-cancer marker (Zhu et al. 2025a, b), we imagine two scenarios, (I) the advantage of a specific signature almost exclusively for liver cancer patients, and (II) the possibility that *ADRA1A* signature could function in other pathological contexts where PCK1 is expressed and relevant, independently of the cancer type, potentially in renal, colorectal, lung and melanoma cancer (Liu et al. 2024).

Since our current strategy is limited to the analysis of transcriptomic data, our future studies will integrate proteomic data aiming to consolidate the functional relationship between α_1A_-adrenergic receptor and the candidate signaling partners. To evaluate a causal relationship, we envision a systematic strategy as the recently described by Zhu and colleagues in which they identified protein markers and therapeutic targets linked to liver cancer risk (Zhu et al. 2025a, b).

Overall, these strategies are currently contributing to optimize medical decisions important in the prognosis and treatments of cancer patients in hepatocellular carcinoma (Shi et al. 2024; Tang et al. 2024; Zhou et al. 2025). Nonetheless, *ADRA1A*-associated transcriptional signatures could be tested in other cancer types to identify a “hepatoma” or “non-hepatoma” profile.

About the potential clinical translation of the *ADRA1A*-associated transcriptional signatures, validation with independent datasets and experimental testing are needed to evaluate these potential markers and whether they may complement current clinicopathological classifications, such as the Barcelona Clinic Liver Cancer (BCLC) and TNM systems. Conceptually, the complementary value of these transcriptional signatures is based on the functionality of their members which are signaling proteins and, as such, represent pharmacological opportunities as potential drug targets in relevant signaling pathways, adding to the value of the aspartate metabolism as a significant process in AMGS. However, their clinical application requires further preclinical and clinical tests.

It is worth mentioning that* β*_2_-adrenergic receptors are generally expressed in hepatocytes. However, subtype expression changes occur during malignant transformation, i.e.,* β*_1_-adrenergic receptors are expressed in cells such as Zajdela hepatoma (Lacombe et al. 1976) and the transplantable ascites variant of rat hepatoma, AS-30D (Garcia-Sainz et al. 1989). In our present work, no change in the α_1A_-adrenergic receptor subtype was detected, but changes in expression, linked to patient survival, might be relevant to understanding the transcriptional regulation during malignancy, prognosis, and eventually treatment.

## Conclusion

In this study we identified three *ADRA1A*-associated signaling signatures statistically linked to more prolonged patient survival. This suggests the intriguing hypothesis that high* α*_1A_-adrenergic receptor expression and signaling attenuates tumor progression in LIHC patients. The first *ADRA1A*-associated transcriptional signature includes five candidates of the intracellular signaling hardware comprising two RhoGEFs, the second incorporates *ADRA1A* and six other GPCRs and the third involves *ADRA1A* and two RTKs. The three proposed signatures showed higher statistical significance linked to more prolonged patient survival than *ADRA1A* alone, pointing to the valuable identification of a group of potential candidates in the* α*_1A_-adrenergic receptor signaling pathways that warrants future investigations to address their functionality and clinical relevance.

## Supplementary Information

Below is the link to the electronic supplementary material.

Supplementary Material 1

## Data Availability

Data included in the text, figures and supplementary information are provided.
