# Supplementary figures and images for: α1A-Adrenergic receptor (ADRA1A) signaling signatures as prognostic biomarkers in liver hepatocellular carcinoma: a bioinformatic analysis
Source: In Silico Pharmacol. 2026 Jul 31;14(2):202. doi: 10.1007/s40203-026-00706-4 (PMC13427700; doi:10.1007/s40203-026-00706-4)

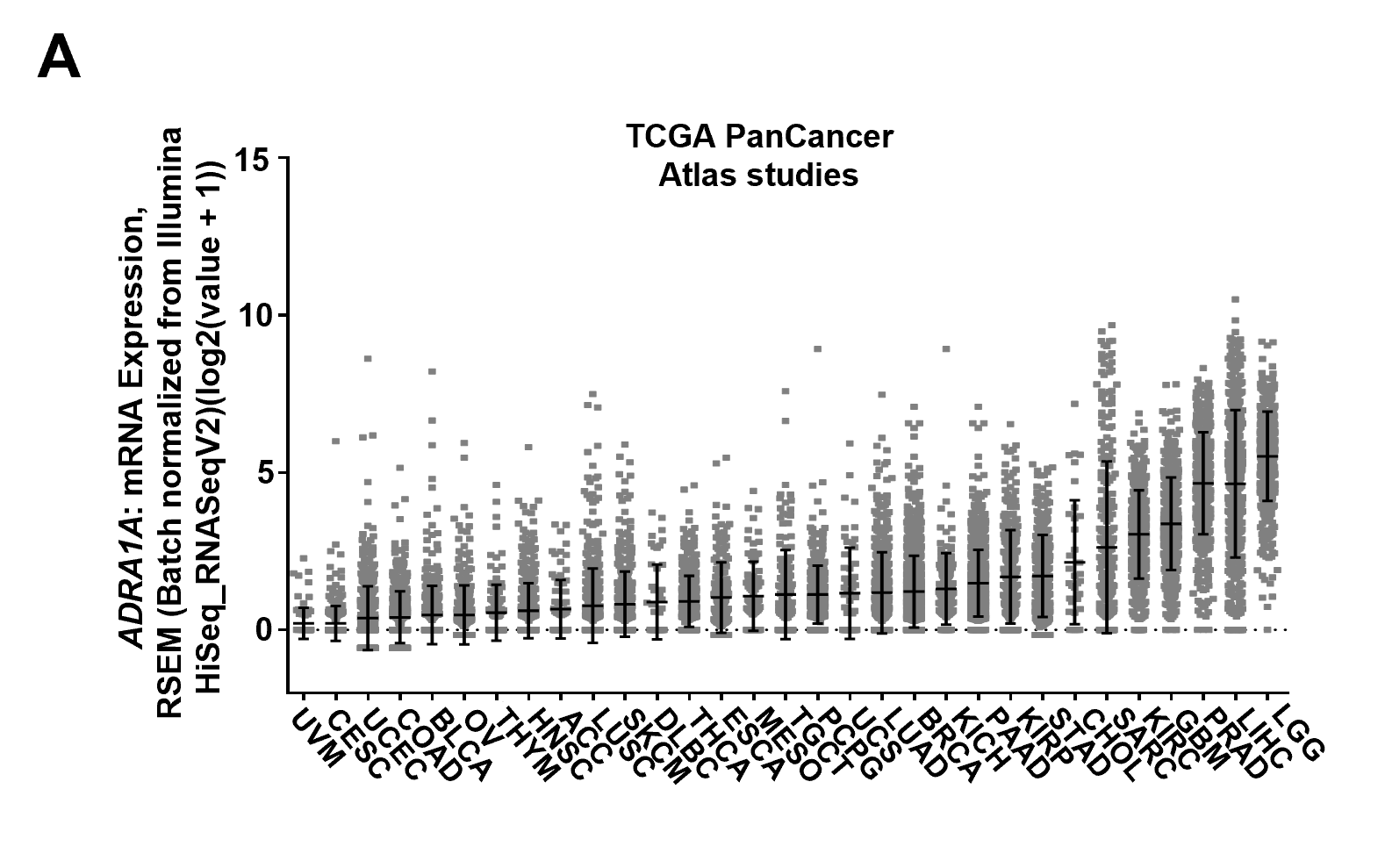

Supplement: Supplementary file 1 — Supplementary Material 1 [file 40203_2026_706_MOESM1_ESM.tif]
